# Feasibility and preliminary efficacy of a combined virtual reality, robotics and electrical stimulation intervention in upper extremity stroke rehabilitation

**DOI:** 10.1186/s12984-021-00851-1

**Published:** 2021-04-14

**Authors:** Nahid Norouzi-Gheidari, Philippe S. Archambault, Katia Monte-Silva, Dahlia Kairy, Heidi Sveistrup, Michael Trivino, Mindy F. Levin, Marie-Hélène Milot

**Affiliations:** 1 School of Physical & Occupational Therapy, McGill University, Montreal, Canada; 2 Interdisciplinary Research Center in Rehabilitation, Montreal, Canada; 3 School of Rehabilitation, University of Montreal, Montreal, Canada; 4 Faculty of Health Sciences, University of Ottawa, Ottawa, Canada; 5 Centre Intégré de santé et services sociaux de Laval, Laval, Canada; 6 School of Rehabilitation, University of Sherbrooke, Sherbrooke, Canada; 7 Research Center on Aging, CIUSSS de l’Estrie-CHUS, Sherbrooke, Canada; 8 Physical Therapy Department, Universidade Federal de Pernambuco, Recife, Brazil

**Keywords:** Stroke, Upper extremity, Rehabilitation, Virtual reality, Robotics, Electrical stimulation

## Abstract

**Background:**

Approximately 80% of individuals with chronic stroke present with long lasting upper extremity (UE) impairments. We designed the perSonalized UPper Extremity Rehabilitation (SUPER) intervention, which combines robotics, virtual reality activities, and neuromuscular electrical stimulation (NMES). The objectives of our study were to determine the feasibility and the preliminary efficacy of the SUPER intervention in individuals with moderate/severe stroke.

**Methods:**

Stroke participants (n = 28) received a 4-week intervention (3 × per week), tailored to their functional level. The functional integrity of the corticospinal tract was assessed using the Predict Recovery Potential algorithm, involving measurements of motor evoked potentials and manual muscle testing. Those with low potential for hand recovery (shoulder group; n = 18) received a robotic-rehabilitation intervention focusing on elbow and shoulder movements only. Those with a good potential for hand recovery (hand group; n = 10) received EMG-triggered NMES, in addition to robot therapy. The primary outcomes were the Fugl-Meyer UE assessment and the ABILHAND assessment. Secondary outcomes included the Motor Activity Log and the Stroke Impact Scale.

**Results:**

Eighteen participants (64%), in either the hand or the shoulder group, showed changes in the Fugl-Meyer UE or in the ABILHAND assessment superior to the minimal clinically important difference.

**Conclusions:**

This indicates that our personalized approach is feasible and may be beneficial in improving UE function in individuals with moderate to severe impairments due to stroke.

**Trial registration:**

ClinicalTrials.gov NCT03903770. Registered 4 April 2019. Registered retrospectively.

**Supplementary Information:**

The online version contains supplementary material available at 10.1186/s12984-021-00851-1.

## Introduction

Approximately 80% of individuals with stroke experience hemiparesis of the upper extremity (UE) [1] leading to chronic impairments such as weakness, loss of motor control, edema, pain and spasticity. These have important consequences for quality of life as impairments in hand and arm function limit participation in activities of daily living [2, 3]. Accordingly, recovery of UE function is seen as highly important by individuals with chronic stroke, caregivers and rehabilitation professionals [4].

According to the Canadian Stroke Best Practices [5], UE rehabilitation should involve the affected limb in “training that is meaningful, engaging, repetitive, progressively adapted, task-specific and goal-oriented”. Advances in rehabilitation technology, in particular robotics, virtual reality (VR) and neuromuscular electrical stimulation (NMES), have been shown to be individually effective for improving UE function of individuals with stroke, through the provision of such repetitive and task-oriented training. Robotic devices can be used to assist individuals who are unable to complete arm movements by themselves [6]. Robotic rehabilitation has demonstrated functional gains in individuals with mild and moderate stroke impairments [7–9]. Likewise, some of our recent work [10] has shown that individuals with severe, chronic stroke can improve their arm range of motion and clinical scores after ten sessions of robotic therapy. However, it should be noted that functional gains in robotic therapy are not greater than those obtained with similar intensity conventional therapy [8]. While the intensity of practice is a determining factor in stroke recovery, higher improvements might have been achieved by robotic therapy if its focus was not only on shoulder and elbow movements, but also on hand function. This may be possible by integrating robotic therapy in a rehabilitation program that also includes other modalities that better target hand function.

VR activities constitute another approach to UE stroke rehabilitation, where patients typically perform movements without physical assistance. Reviews examining the use of VR for the improvement of UE function show promising results [11, 12]. In our view, VR could consolidate the UE functional gains obtained through robotic rehabilitation. While most VR activities typically focus on shoulder and elbow movements, some recent technical advances now allow the inclusion of hand movements as well. Specifically, the Microsoft Kinect version 2, used to track movements in VR, can detect hand opening and closing in addition to shoulder, elbow and wrist movements. These capabilities have been included in a new rehabilitation application, targeting UE reaching and grasping movements [13], which was part of our rehabilitation approach.

Electromyographically (EMG)-triggered NMES is a muscle stimulation modality that has been used to facilitate motor recovery of the hand after stroke [14]. The individual with stroke needs to activate the muscle(s) volitionally to trigger the NMES [15]. Thus, EMG-triggered NMES provides wrist and/or finger extension time-locked to the cognitive movement intent to actively extend the wrist and open the hand, making the training ecological and functionally relevant. EMG-triggered NMES has been shown to improve voluntary activation of isolated muscles, particularly in task-specific patterns [16].

While advances in robotics, VR and NMES have led to new treatment modalities targeting UE function post-stroke, further progress is needed for these technologies to have a true impact. Despite numerous studies attempting to identify the most effective rehabilitation interventions, post-stroke UE recovery remains disappointing [17] with sensorimotor deficits persisting in a large proportion of stroke survivors for more than 6 months (up to 62% [18]). Improvements in clinical scores have been small and often fail to meet the criteria for minimal clinically important differences (MCID) [19]. While most of the recent clinical trials have failed to demonstrate improvements on UE function that favour new interventions such as robotics or VR, over conventional, dose-matched therapy [20], combination of different modalities may have a greater impact on stroke recovery than each individual modality alone [21]. Thus, there is a need to look beyond the ‘one-size-fits-all’ approach, where a single UE modality is applied to a group of post-stroke individuals. Another possible reason for the relatively small gains in UE function, and in particular the low gains in hand function [17], is that an individual’s potential for recovery is not always considered [20]. In clinical practice, therapists typically prescribe UE exercises to their clients based on initial clinical measures, which turn out to be poor predictors of future UE function [22]. However, assessing the integrity of the affected corticospinal tract (CST), by means of motor evoked potentials (MEPs) elicited by non-invasive transcranial magnetic stimulation (TMS), was found to strongly predict the changes in UE function that could be elicited by rehabilitation [23]. In particular, the work by Milot et al. [24] showed that amongst several brain measures (e.g., magnetic resonance imaging, diffusion tensor imaging), baseline MEP amplitude was the best predictor of the response to robotic training of the affected UE in chronic stroke survivors. The presence of a MEP indicates that the CST, linking the motor areas of the brain to the hand musculature, is at least partially preserved.

Considering that (1) an individualized intervention to post stroke UE rehabilitation is desirable, (2) CST integrity is a strong predictor of hand function recovery, and (3) combination of different modalities may have a greater impact on stroke recovery than each individual modality alone, our proposed approach was to combine multiple modalities in an individualized intervention, tailored to each stroke participant’s functional status and recovery potential. Recovery may be enhanced by first assessing CST integrity in order to determine the potential for recuperating hand function, and then combining multiple purposefully selected combinations of modalities to target motor deficits of each individual. Specifically, our perSonalized UPper Extremity Rehabilitation (SUPER) program included: (1) robotic activities to work on physically assisted UE reaching movements; (2) VR activities to work on unassisted reaching and grasping movements; and (3) NMES to facilitate hand opening and closing movements. The frequency of incorporation of each modality during the intervention was determined according to the individual’s potential for hand recovery. Our objectives were to determine the feasibility and the treatment effect of the SUPER program in individuals with moderate/severe chronic stroke. Our hypotheses were that (1) the SUPER program would be feasible in terms of process, resources, management and safety indicators and (2) stroke participants with a low potential for hand recovery would benefit from a shoulder/elbow-centered intervention, while those with a high potential would benefit from an intervention involving the whole arm.

## Methods

### Study design

We used a pre/post, single-subject design with multiple baselines (AAAB). Baseline assessments were repeated three times within one week (T0-A, T0-B, T0-C), while post intervention took place immediately following the end of the SUPER program (T1). All assessments were conducted by a therapist at each site blinded to participant allocation and to the study objectives.

### Participants

We recruited 30 individuals with chronic stroke from the list of potential subjects who were a prior client at two rehabilitation centers in Quebec, Canada: CISSS Laval–Jewish Rehabilitation Hospital and CIUSSS de l’Estrie–CHUS. The inclusion criteria were: (1) ischemic or hemorrhagic stroke; (2) moderate to severe UE impairment (score between 2 and 4 out of 7 on the Chedoke-McMaster Arm and Hand Scales); (3) at least 3 months post stroke; and (4) no longer receiving rehabilitation services. Exclusion criteria were factors that may have limited participation or understanding of instructions: (1) medical instability; (2) marked cognitive deficits (mini-cog [25] score < 3); (3) uncorrected visual impairments; (4) shoulder pain (score ≥ 4 on the visual analog scale); (5) severe spasticity in wrist flexors (score > 3 on the Modified Ashworth Scale); (6) contraindications to NMES. The participant’s potential for UE recovery was first determined by TMS to measure CST integrity (see below).

### Potential for hand function recovery

The integrity of the affected CST was assessed using MEPs elicited by TMS, as per the Predict Recovery Potential (PREP) procedure [26]. Surface electrodes were placed in a belly-tendon montage over the affected first dorsal interosseous muscle and the extensor carpi radialis. TMS was performed with the figure-of-eight coil held tangentially over the primary motor cortex (C3/C4 according to 10–20 system). The intensity was set at 100% of the stimulator output (Magstim 200^2^, Magstim Company, Dyfed, UK). If needed, a different scalp location was also tried (2 cm posterior to C3/C4). MEP was considered absent if no response higher than a peak-to-peak amplitude of 50 µV could be obtained in either muscles after 3 stimuli at each location. The presence or absence of a MEP was used to classify participants as having a high or a low potential for the recovery of hand function, respectively. In participants where TMS was contraindicated (due to medical conditions such as epilepsy), the CST integrity was evaluated through manual muscle testing of shoulder abductors and finger extensors [26].

### SUPER intervention

Participants received a total of 12, 1-h SUPER intervention sessions, at 3 times per week for 4 weeks. For participants with a low recovery potential for hand function (*shoulder* group), the SUPER intervention consisted of robot-assisted reaching movements only. For those with a good recovery potential (*hand* group), the SUPER intervention included VR activities combined with NMES, in addition to robotic therapy; the participants in this group first attended robotic therapy and then VR/NMES activity in each session. The weekly amounts of robotics and VR/NMES sessions were based on the ability of participants to produce unassisted reaching movements with their affected arm against gravity.

**Fig. 1:**
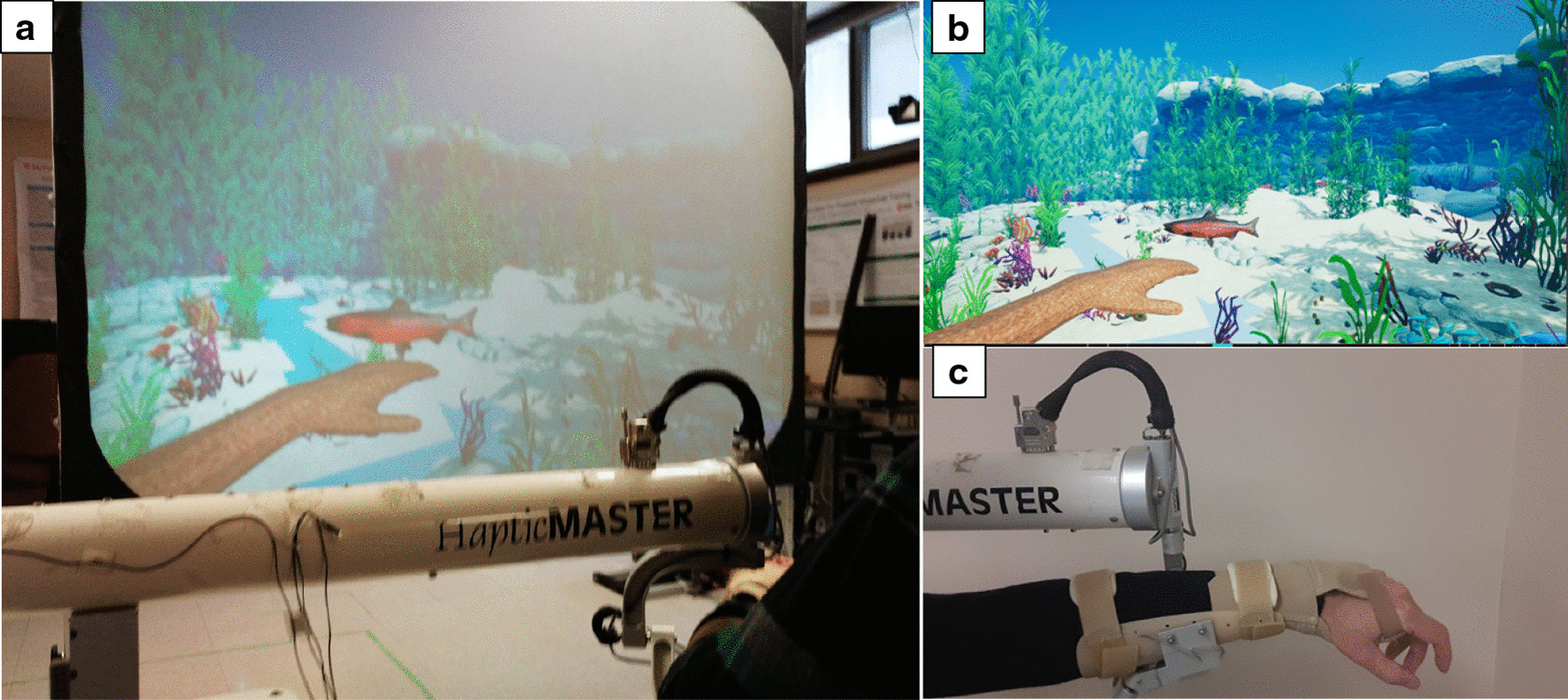
**a** Overall view of robotic rehabilitation system. **b** Screen capture of virtual scene. **c** Arm/hand support system

The robot-assisted activity consisted of a virtual underwater fishing game, where participants had to reach for targets placed at their maximal voluntary arm extension (Fig. 1). The robot provided weight support to the arm by creating a dynamic virtual support surface between the starting location and the reaching target. If a participant was unable to reach for a target on their own, the robot could prevent backwards movement (movement away from the target). It could also provide a pushing force to physically assist the movement. The type of robotic assistance (if any) was decided upon by the attending therapist. At the end of each trial, participants received feedback on the percentage of the movement that was performed independently (without robotic assistance) and were encouraged to increase that number as much as possible.

**Fig. 2:**
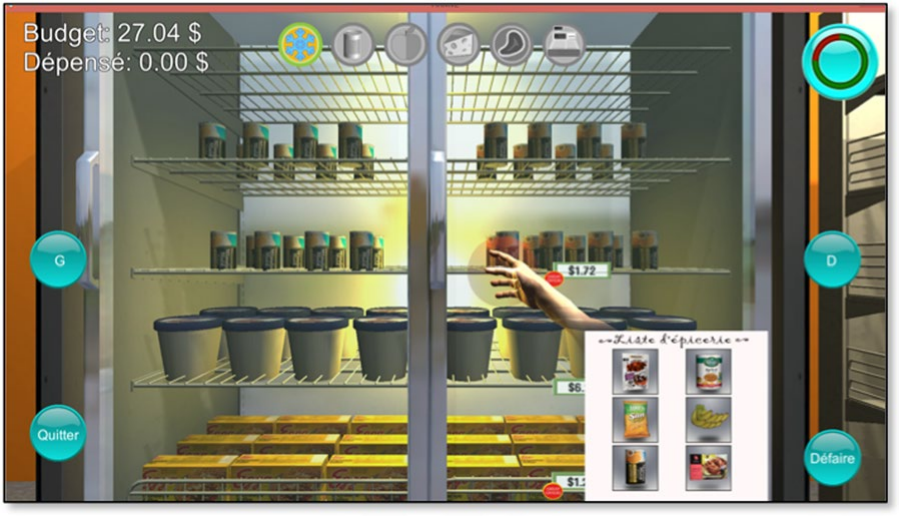
Virtual Reality grocery shopping activity

For VR, we used a reach and grasp VR activity based on a grocery shopping task (Fig. 2). Participants interacted with the VR activity through arm reaching movements and hand opening/closing movements, which were recorded by a Kinect V2 camera (Microsoft, USA). Participants were required to reach for grocery items placed on a shelf, grab them by closing their hand, bring them near their body and release them in their virtual shopping cart by opening their hand.

Thirty minutes of EMG-triggered NMES treatment was also incorporated in the VR sessions of reaching and grasping for participants in the hand group. Two-inch diameter surface electrodes attached to the NMES device were placed over the motor points of the Extensor Digitorum Communis for activating finger/wrist extension. Stimulation intensity (pulse amplitude) was set to produce maximal finger extension without discomfort. The electrical stimulation was triggered when the instrument detected an EMG signal exceeding a pre-set threshold, determined earlier during practice. The muscle stimulation was triggered when the participant attempted to grasp an object on the computer screen during VR activities. Thus, as the participant attempted to reach for an object, the stimulator picked up the EMG signal generated by the attempted wrist extension movements and stimulated the extensors to assist with hand opening during the reaching attempt. At the end of the reach, the stimulator was turned off until the next attempt.

To summarize, participants in the shoulder group received 60 min of robotic therapy. Some, who were able to move their arm against gravity, also received the VR intervention (e.g., 30 min of robot therapy and 30 min of VR therapy). Participants in the hand group received approximately 30 min of robotic therapy, followed by 30 min of VR therapy combined with NMES.

### Feasibility indicators

Our feasibility criteria are listed in Table 1. These included indicators related to process, resources, management and safety. For perceived satisfaction, we used a questionnaire rating enjoyment, perceived performance during the activity and perceived value of the activity. Questions were answered on a 7-point (pt) Likert scale, ranging from 1 (not at all true) to 7 (very true). The questionnaire was administered for both activities (robotics and VR/NMES), depending on the activity in which each participant was engaged.

**Table 1:** Feasibility indicators

Feasibility component	Outcome measure	Criterion for success	Study results
Process			
Recruitment rate	Number of Part. recruited	2 part./month/site	1.9 part./month/site
Consent rate	% part. consenting	> 65% of part	93%
Retention rate	% part. completing study	> 85% complete T1	93%
Perceived satisfaction	7-pt Likert scale	> 85% report perceived benefits	100%
Resources			
Adherence rate	Attend ≥ 10/12 intervention sessions	> 85% of subjects	100% attended 12 sessions
Session length	Evaluations < 2 h	> 85% of evaluations	100%
Intervention burden	Interventions < 1.15 h	> 85% of sessions	89.3%
Management			
Participant processing time	Time initial contact to enrolment	Mean time < 10 days	100%
Treatment			
Safety (evaluation)	Adverse events	No adverse event	No adverse event
Safety (intervention)	Adverse events	No adverse event	No adverse event

Part.: Participants

### Treatment outcomes

The primary outcomes were: (1) the Fugl-Meyer Assessment [27], upper extremity section (FMA-UE), a performance-based measure of UE impairment describing motor recovery. (2) The ABILHAND [28], a questionnaire to assess active function of the hands and upper limbs in daily living tasks. Both measurement tools have excellent psychometric properties [29, 30].

As secondary measures, we used: (3) the Motor Activity Log, 14-item version (MAL-14), which rates self-reported quality and frequency of use of the UE in 14 everyday tasks [31, 32]; (4) the Stroke Impact Scale (SIS), a stroke-specific health status measure featuring 33 items capturing daily activities grouped in 8 sub-scales (strength, memory, mood, communications, activities, mobility, hand function and quality of life) [33]; (5) the Box and Blocks Test (BBT), a measure of gross motor dexterity; (6) Hand Grip Strength of the affected UE, measured with a dynamometer.

### Analyses

For the efficacy outcomes (primary and secondary), our criteria for quantifying the importance of a difference was based on the established MCID of 5.25 for FMA-UE [34], 0.26 for ABILHAND [35] and 1 for MAL [36]. For the SIS, analyses focused on three specific subscales: strength (SIS-Strength; MCID: 9.2); activities of daily living (SIS-ADL; MCID: 5.9) and hand tasks (SIS-Hand; MCID: 17.9) [37]. For each outcome, we first computed the difference between the post-evaluation measure and the mean of the three baseline measures. We then counted the number of participants who displayed a change greater than the MCID.

We defined *responders* as participants who displayed a change with respect to baseline that was larger than the MCID, in either of the primary outcomes (FMA-UE or ABILHAND). Participants who did not improve in either of the primary outcomes were classified as *non-responders*. As a secondary analysis, we compared responders to non-responders with respect to demographic characteristics (age, stroke onset, sex, stroke side) and baseline outcome values. Groups were compared using either independent t-tests (for continuous variables) or χ^2^ (for proportions). The average change between pre- and post-intervention are also reported and were compared within each group using the Wilcoxon signed rank test.

## Results

### Participants

A total of 30 participants were recruited between July 2018 and February 2019. Of these, 28 completed all sessions of the SUPER intervention and all the evaluations. All the subjects were tested using PREP algorithm except two of them for whom manual muscle testing of shoulder abductors and finger extensors were used. Two participants did not initiate the intervention due to medical or personal reasons unrelated to the study and were therefore excluded from the analyses. As can be seen in Table 2, after classification with the PREP algorithm, 10 participants were included in the hand group (good CST functional integrity), while 18 where in the shoulder group (poor CST functional integrity). Both groups were similar in terms of sex (χ^2^; p = 0.83), age (t-test; p = 0.52) and stroke onset (t-test; p = 0.78). Baseline FMA-UE, BBT and ABILHAND were understandably lower in the shoulder group than in the hand group (t-test; p < 0.05).

**Table 2:** Participants’ demographics and baseline characteristics

	Hand group	Shoulder group
N	10	18
Sex (F/M)	4/6	6/12
Age (years) (range)	61.9 (38.3 to 76.3)	65.6 (56.3 to 76.8)
Stroke onset (years) (range)	5.7 (0.8 to 25.8)	6.2 (0.6 to 24.2)
FMA-UE (/66) (range)	41.2 (26 to 54)	13.9 (8 to 23)
BBT (n blocks) (range)	12.7 (0 to 33)	0.2 (0 to 3)
ABILHAND (-6 to 6) (range)	1.9 (0.2 to 3.9)	0.2 (− 2.4 to 3.3)

*F* female, *M* male, *FMA-UE* Fugl-Meyer Assessment-Upper Extremity section, *BBT* box and blocks test

### Feasibility indicators

Results for the feasibility indicators are shown in Table 1. In terms of resources, most of the indicators were met. Our recruitment rate was 30 participants over 8 months, or an average of 1.9 participant/site/month, slightly below our target of 2. The retention rate was 93%, greater than our criterion of retention success of 85%, and perceived satisfaction was high, i.e. 100%. Indeed, all participants strongly agreed with questions such as “I enjoyed doing this activity very much” and “I believe this activity could be of value to me”, both for the robotics and VR/NMES activities. All the resource and management indicators were met. All participants (100%) attended the full 12 sessions. Average session length was 54 min, with only 10.7% of sessions lasting more that 1 h and 15 min. Importantly, no adverse effect was reported, either during the evaluations or during the interventions.

### UE Recovery

**Fig. 3:**
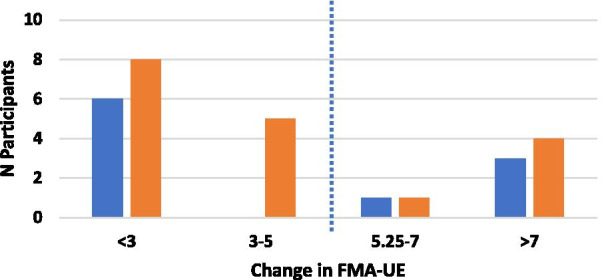
Changes in Fugl Meyer Assessment-Upper Extremity from baseline for the hand (blue bars) and shoulder (orange bars) groups. Dotted line indicates Minimal Clinically Important Difference

As illustrated in Fig. 3, nine participants showed changes exceeding the MCID of 5.25 pt for the FMA-UE. Of these, four were in the hand group and five in the shoulder group, representing a similar proportion within the two groups (40% and 28%, respectively; χ^2^ test; p < 0.5). Considering all participants, i.e. responders and non-responders, the hand group average change was 4.5 pt (standard error (SE) = 2.6, Z = − 1.84, p = 0.066) and the shoulder group change was 3.4 pt (SE = 0.8, Z = − 3.10, p = 0.002).

### Dexterity

**Fig. 4:**
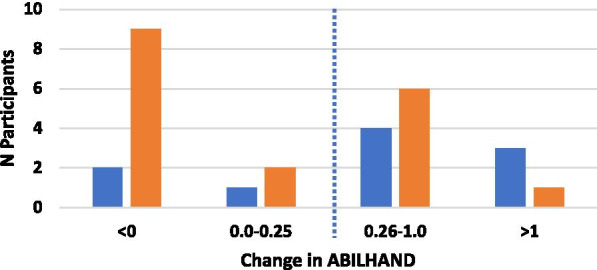
Changes in ABILHAND from baseline for the hand (blue bars) and shoulder (orange bars) groups. Dotted line indicates Minimal Clinically Important Difference

For the ABILHAND (Fig. 4), seven participants in the shoulder group and seven in the hand group showed improvements greater than the MCID following the intervention (39% and 70%, respectively). The proportion of participants with improvement in the ABILHAND in the shoulder and hand groups was not significantly different (χ^2^ test; p < 0.1). The average hand group change was 0.7 pt (SE = 0.27, Z = − 2.09, p = 0.037) and the shoulder group change was 0.16 pt (SE = 0.13, Z = − 0.98, p = 0.327).

### Secondary outcomes

Almost none of the participants in the shoulder group were able to perform the BBT with their affected hand, during baseline evaluation and no changes were observed after the SUPER intervention in this outcome. In the hand group, there were no differences in performance of the BBT with the affected hand, before and after the intervention, that exceeded the MCID.

Three participants showed improvements in the MAL-14 superior to the MCID, following the SUPER intervention. All three were in the hand group.

**Fig. 5:**
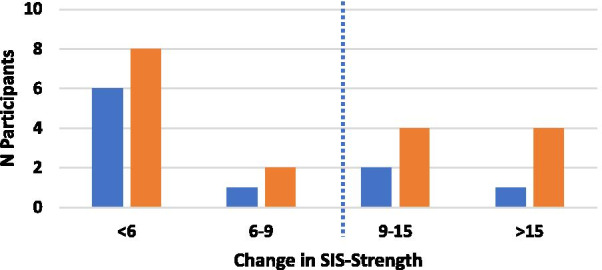
Changes in Stroke Impact Scale-Strength from baseline for the hand (blue bars) and shoulder (orange bars) groups. Dotted line indicates Minimal Clinically Important Difference

**Fig. 6:**
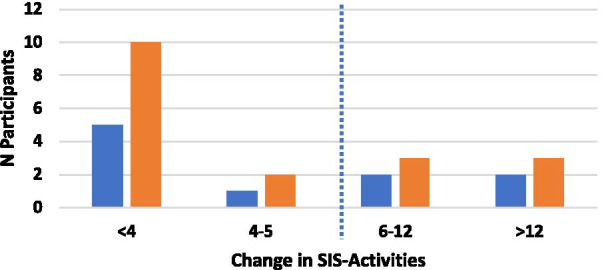
Changes in Stroke Impact Scale-Activities from baseline for the hand (blue bars) and shoulder (orange bars) groups. Dotted line indicates Minimal Clinically Important Difference

For eleven participants, the SUPER intervention resulted in improvements in the SIS-Strength subscale (four in the hand group and five in the shoulder group) greater than the MCID (Fig. 5). The average change for the hand group was 7.9 pt (SE = 4.2, Z = − 2.04, p = 0.041) and for the shoulder group was 8.1 pt (SE = 2.3, Z = − 2.79, p = 0.005). Likewise, as seen in Fig. 6, nine participants showed improvements in the SIS-ADL subscale (three in the hand group and six in the shoulder group). The average change for the hand group was 6.2 pt (SE = 2.5, Z = − 2.37, p = 0.018) and for the shoulder group was 4.0 pt (SE = 1.7, Z = − 2.02, p = 0.043). As for the SIS-Hand subscale, only one participant (hand group) displayed changes above MCID. The average change for the hand group was 8.8 pt (SE = 2.7, Z = − 2.31, p = 0.021) and for the shoulder group was 0.3 pt (SE = 0.9, Z = − 0.41, p = 0.686).

None of the participants showed any improvement in grip strength following the SUPER intervention.

### Responders and non-responders

Taken together, eight participants in the hand group (80%) were classified as responders to the SUPER intervention, as their improvement in the FMA-UE, the ABILHAND, or both, was higher than the respective MCID. For the shoulder group, ten participants were classified as responders (56%). Table 3 shows the demographics and baseline scores of some baseline measures for responders and non-responders to the SUPER intervention. Note that, as there were only two non-responders in the hand group, both the shoulder and hand groups were pooled together in this table. Responders and non-responders did not differ in terms of age, sex and in the non-physical subscales of the SIS (memory, mood, communications and quality of life). The non-responders were in the more chronic stage post-stroke compared to the responders (stroke onset tended to be higher), but the difference was not significant (t-test; p < 0.08). Likewise, the proportion of participants with right-side hemiparesis was higher in the non-responders (60%, as compared to 33% in the responders), but these proportions were not significantly different (χ^2^ test; p < 0.19).

**Table 3:** Differences in demographics and baseline scores between responders and non-responders

	Responders (n = 18)	Non responders (n = 10)	p-value
Age	63.5 ± 9.5	66.6 ± 8.5	0.58
Stroke onset	4.2 ± 3.2	9.7 ± 11.8	0.08
SIS-memory	81.6 ± 20.7	90.2 ± 11.8	0.24
SIS-mood	61.3 ± 15.6	68.1 ± 16.6	0.29
SIS-communications	80.9 ± 21.9	86.1 ± 20.5	0.54
SIS-QOL	57.7 ± 18.0	47.8 ± 25.2	0.24
Prop. female	0.33	0.40	0.42
Prop. right hemi	0.33	0.60	0.19
Prop. hand Gr	0.80	0.20	
Prop. shoulder Gr	0.56	0.44	

*SIS* Stroke Impact Scale, *QOL* Quality of Life, *Prop*. proportion, *Gr* Group; ±  mean ± standard deviation

## Discussion

In this study, we established the feasibility and measured the preliminary efficacy of the SUPER intervention, combining robotics, VR and NMES, to improve UE function in individuals with chronic stroke. The intervention was personalized according to each participant’s potential for hand recovery, as assessed by the PREP algorithm. Our results demonstrated that the SUPER intervention was safe and well received by participants, who viewed both the robotics and VR activities as enjoyable and potentially beneficial. This was also confirmed by the high retention rate (93%) and the fact that all participants completed their 12 intervention sessions.

Our results also indicated that 18 participants (80% of the hand group (8 out of 10) and 56% of the shoulder group (10 out of 18)) improved in terms of our primary outcomes (FMA-UE or ABILHAND) following the SUPER intervention. Most also improved in other measures related to the performance of UE activities (SIS or MAL-14). This supports other studies stating that motor recovery is still possible in the chronic phase of a stroke [38] and that even severely impaired stroke survivors can gain of long-term rehabilitation [39]. However, few or no participants improved in measures specific to hand strength or dexterity (BBT, grip strength, SIS-Hand). While this was expected for participants in the shoulder group, who did not perform any hand related exercises, it seems the SUPER program only had limited impact on hand function for participants in the hand group as well. This could have been due to an insufficient number of trials or a lack of variability in hand tasks. Future studies could incorporate another VR/NMES and/or robotic task (e.g., grasping objects of different shapes and sizes) to address this lack of hand function gains following training.

Our analyses did not reveal any significant demographic/baseline differences (depicted in Table 3) between participants who responded to the SUPER intervention and those who did not. While there seems to be differences between responders and non-responders in terms of stroke onset time, level of impairment (i.e., hand group or shoulder group) and stroke side, these were not significant. While initial upper limb impairment and function level has been identified as a strong predictor of stroke UE recovery, results about stroke onset time and stroke side have been equivocal [40]: some trials have linked left-sided stroke and longer stroke onset times with poorer UE outcomes, but others have not [40]. Nevertheless, participants in the hand and shoulder groups improved in UE function in similar proportions, indicating that our personalized/multi-modal approach was beneficial to most. This suggests that a personalized intervention, focusing on either shoulder/elbow or the whole UE depending on the level of CST integrity, may be more appropriate than a ‘one-size-fits-all’ approach where all participants receive the same intervention. The benefits of a personalized/multi-modal intervention are that participants can spend more time practicing tasks that target their specific impairments and/or for which they may have the most potential for recovery. Such an approach is also closer to what is done during stroke rehabilitation, where clinicians use clinical reasoning and employ a combination of tasks and activities, although not necessarily tailored to each of their client’s potential, to promote recovery. One limitation of our study design, however, is that we cannot determine the relative importance of one or another component of the SUPER intervention.

While 32% of our participants improved FMA-UE and 50% improved ABILHAND scores greater than the MCID value of each measure, 82% showed improvements in at least one of the outcome measures (FMA-UE, ABILHAND, MAL-14 or SIS) greater than the MCID values. It is, however, difficult to explain an improvement in activities involving the UE, as measured by the MAL and SIS, without a decrease in the underlying impairment level, as measured by the FMA-UE. We prefer therefore to keep the more conservative estimate of 64% of participants showing improvements in the FMA-UE or ABILHAND, following the SUPER intervention.

Another limitation of the current study is the absence of a control group making it harder to attribute improvements in UE function specifically to the SUPER intervention. For example, as our participants were all in the chronic stage of stroke, some may have suffered from physical deconditioning of their affected UE due to disuse. A rehabilitation intervention based on usual care (e.g., exercises and ADL tasks) may have yielded similar results. Indeed, research indicates that both robotics and VR lead to similar improvements in UE function as conventional therapy (not using rehabilitation technology modalities) when practice time is matched [8]. In addition, we did not assess if gains in UE function were maintained in the months following the intervention. A follow-up study could include a control group receiving time-matched conventional therapy and reassessment after 3–6 months.

## Conclusions

Approximately two thirds (64%) of chronic stroke participants improved their UE function, following 12 sessions of the SUPER intervention, which combined robotics, VR and NMES. Participants with both good and poor potential for recovery of hand function displayed clinically important improvements, indicating that our personalized intervention is suitable for individuals with moderate, as well as with severe limitations due to stroke. In addition, the intervention was feasible and safe. Future work will look at how the SUPER intervention could be improved to better target hand function and to determine if functional gains are maintained after 3 to 6 months.

## Supplementary Information

**Additional file 1.** Supplementary Table A: Changes in score between baseline and post-treatment measurements. The results are illustrated in Figures 3 to 6.

## Data Availability

The datasets generated during and/or analysed during the current study are not publicly available due to IRB restrictions, but are available from the corresponding author on reasonable request.

## Abbreviations

BBT: Box and blocks test
CST: Corticospinal tract
FMA-UE: Fugl-Meyer Assessment—upper extremity
MAL: Motor activity log
MCID: Minimal clinically important difference
NMES: Neuromuscular electrical stimulation
SIS: Stroke Impact Scale
SUPER: PerSonalized UPper Extremity Rehabilitation
TMS: Transcranial magnetic stimulation
VR: Virtual reality
UE: Upper extremity
