# Supplementary material for: Feasibility and preliminary efficacy of a combined virtual reality, robotics and electrical stimulation intervention in upper extremity stroke rehabilitation
Source: J Neuroeng Rehabil. 2021 Apr 14;18:61. doi: 10.1186/s12984-021-00851-1 (PMC8045249; doi:10.1186/s12984-021-00851-1)
Supplement: Supplementary file 1 — Additional file 1. Supplementary Table A: Changes in score between baseline and post-treatment measurements. The results are illustrated in Figures 3 to 6. [file 12984_2021_851_MOESM1_ESM.docx]

**Supplementary Table A:** Changes in score between baseline and post-treatment measurements. The results are illustrated in Figures 3 to 6.

| **Group** | **FMA-UE** | **ABILHAND** | **MAL AOU** | **MAL QOM** | **SIS Strength** | **SIS Memory** | **SIS Mood** | **SIS Communicate** | **SIS Activities** | **SIS Mobility** | **SIS Hand** | **SIS QoL** | **SIS Recovery** | **SIS Physical** |
| --- | --- | --- | --- | --- | --- | --- | --- | --- | --- | --- | --- | --- | --- | --- |
| Hand | 10.0 | 0.6 | 0.60 | 1.35 | 3.1 | 1.8 | 6.9 | -14.3 | 0.1 | -1.4 | -5.0 | -37.5 | 2.5 | -0.8 |
| Hand | 12.0 | -0.7 | -0.09 | -0.48 | 3.1 | 0.0 | 12.5 | 0.0 | 0.0 | 12.5 | 15.0 | -7.8 | -2.5 | 7.7 |
| Hand | 3.0 | 0.9 | 1.31 | 1.44 | 4.2 | 4.8 | 0.9 | -3.6 | 15.0 | 0.0 | 3.3 | 12.5 | 3.3 | 5.6 |
| Hand | 3.0 | 1.5 | 1.52 | 1.06 | -6.3 | 0.0 | -11.1 | 1.2 | 4.2 | 4.6 | 6.7 | 16.7 | 5.0 | 2.3 |
| Hand | 0.0 | -0.1 | 0.79 | 0.45 | 8.3 | 13.1 | 12.0 | 31.0 | 11.7 | 7.4 | 3.3 | 6.3 | 13.3 | 7.7 |
| Hand | 20.7 | 2.2 | -0.59 | 0.87 | 41.7 | 4.8 | -13.9 | 16.7 | 22.5 | 22.2 | 18.3 | 17.7 | -3.3 | 26.2 |
| Hand | -10.5 | 0.0 | -0.16 | -0.10 | 12.5 | -3.6 | 0.0 | 0.0 | 7.5 | -9.7 | 15.0 | -7.6 | 30.0 | 6.3 |
| Hand | 1.3 | 1.4 | 0.86 | 0.64 | -2.1 | 8.3 | 13.0 | -3.6 | 0.8 | 4.6 | 20.0 | 0.0 | 6.7 | 5.8 |
| Hand | 5.3 | 0.7 | 0.14 | 0.06 | 2.1 | 0.0 | 0.9 | 0.0 | 0.0 | 9.3 | 11.7 | -10.4 | 0.0 | 5.8 |
| Hand | 0.3 | 0.4 | 0.00 | 0.00 | 12.5 | 0.0 | 22.2 | 7.1 | 0.0 | 0.0 | 0.0 | 3.1 | 5.0 | 3.1 |
| **Average** | 4.5 | 0.70 | 0.44 | 0.53 | 7.9 | 2.9 | 4.4 | 3.5 | 6.2 | 5.0 | 8.8 | -0.7 | 6.0 | 7.0 |
| **Std. Err.** | 2.6 | 0.27 | 0.22 | 0.21 | 4.2 | 1.6 | 3.6 | 3.9 | 2.5 | 2.8 | 2.7 | 5.2 | 3.1 | 2.3 |
| **Group** | **FMA-UE** | **ABILHAND** | **MAL AOU** | **MAL QOM** | **SIS Strength** | **SIS Memory** | **SIS Mood** | **SIS Communicate** | **SIS Activities** | **SIS Mobility** | **SIS Hand** | **SIS QoL** | **SIS Recovery** | **SIS Physical** |
| Shoulder | -0.7 | 1.0 | -0.06 | 0.02 | 27.1 | 7.1 | -13.4 | 8.3 | 15.8 | 0.0 | 3.3 | -1.0 | 15.0 | 11.6 |
| Shoulder | 8.3 | 0.7 | 0.38 | 0.29 | 20.8 | 0.0 | 4.6 | 0.0 | -9.2 | 1.9 | 5.0 | 0.0 | 10.0 | 4.6 |
| Shoulder | 0.7 | 0.8 | 0.14 | 0.12 | 8.3 | 6.0 | 5.6 | -19.0 | 0.8 | -7.4 | 0.0 | -13.5 | 5.0 | 0.4 |
| Shoulder | -0.7 | -0.2 | -0.17 | -0.05 | 4.2 | -13.1 | 15.2 | 1.2 | 1.7 | 0.9 | 0.0 | 11.5 | 10.0 | 1.7 |
| Shoulder | -3.0 | -0.2 | -0.14 | -0.05 | -8.3 | -7.1 | -10.2 | -7.1 | 9.2 | 8.3 | -1.7 | 3.0 | 3.0 | 1.9 |
| Shoulder | 2.0 | -0.2 | -0.04 | -0.10 | 10.4 | 27.4 | 4.2 | 4.8 | 5.6 | 5.2 | 0.0 | -5.2 | 10.0 | 5.2 |
| Shoulder | 4.0 | 0.3 | 0.03 | -0.08 | 0.0 | 2.4 | -7.4 | 6.0 | 5.0 | 4.6 | 0.0 | 22.9 | 0.0 | 2.4 |
| Shoulder | 2.0 | -0.1 | 0.00 | 0.00 | 12.5 | -1.2 | 0.0 | 2.4 | -5.8 | -6.5 | 0.0 | -20.8 | -16.7 | 0.0 |
| Shoulder | 1.7 | 0.1 | 0.00 | 0.07 | 0.0 | 7.1 | 11.1 | -4.8 | -1.7 | 24.1 | 0.0 | -22.9 | 0.0 | 5.6 |
| Shoulder | 7.3 | -0.3 | -0.01 | -0.05 | -6.3 | 4.8 | -7.4 | 7.1 | 2.5 | -0.9 | 0.0 | -3.1 | 10.0 | -1.2 |
| Shoulder | 5.0 | 0.8 | 0.05 | 0.05 | 12.5 | 0.0 | 5.6 | 3.6 | 10.8 | -14.8 | 10.0 | -47.9 | -1.7 | 4.6 |
| Shoulder | 5.0 | -0.1 | 0.21 | 0.15 | 2.1 | -6.0 | 2.8 | -7.1 | 0.0 | -0.9 | 0.0 | -7.3 | 20.0 | 0.3 |
| Shoulder | 8.0 | -1.0 | 0.17 | 0.17 | 22.9 | 7.1 | 11.1 | 11.9 | 15.8 | 24.1 | 0.0 | 27.1 | 10.0 | 15.7 |
| Shoulder | 3.7 | -0.2 | 0.38 | 0.31 | 4.2 | 0.0 | 0.0 | 1.2 | 0.0 | 0.0 | -11.7 | 1.0 | 1.7 | -1.9 |
| Shoulder | 8.0 | 0.2 | 0.10 | 0.10 | 6.3 | 32.1 | 15.7 | 4.8 | 14.2 | 18.5 | 0.0 | 3.1 | 6.7 | 9.7 |
| Shoulder | 0.0 | -0.5 | 0.42 | 0.33 | 0.0 | 9.5 | -1.9 | 3.6 | -1.7 | 2.8 | 0.0 | 8.3 | 0.0 | 0.3 |
| Shoulder | 4.0 | 1.1 | 0.07 | 0.07 | 16.7 | 4.8 | 3.7 | 0.0 | -0.8 | -3.7 | 0.0 | -19.8 | 0.0 | 3.0 |
| Shoulder | 5.3 | 0.5 | 0.21 | 0.21 | 12.5 | 0.0 | 1.8 | 3.6 | 9.3 | 5.0 | 0.0 | 10.7 | 10.0 | 4.4 |
| **Average** | 3.4 | 0.16 | 0.10 | 0.09 | 8.1 | 4.5 | 2.3 | 1.1 | 4.0 | 3.4 | 0.3 | -3.0 | 5.2 | 3.8 |
| **Std. Err.** | 0.8 | 0.13 | 0.04 | 0.03 | 2.3 | 2.6 | 1.9 | 1.7 | 1.7 | 2.4 | 0.9 | 4.2 | 1.9 | 1.1 |
| **All** | **FMA-UE** | **ABILHAND** | **MAL AOU** | **MAL QOM** | **SIS Strength** | **SIS Memory** | **SIS Mood** | **SIS Communicate** | **SIS Activities** | **SIS Mobility** | **SIS Hand** | **SIS QoL** | **SIS Recovery** | **SIS Physical** |
| **Average** | 3.8 | 0.35 | 0.22 | 0.24 | 8.0 | 3.9 | 3.0 | 2.0 | 4.8 | 4.0 | 3.3 | -2.2 | 5.5 | 4.9 |
| **Std. Err.** | 1.0 | 0.13 | 0.09 | 0.08 | 2.1 | 1.7 | 1.8 | 1.7 | 1.4 | 1.8 | 1.3 | 3.2 | 1.6 | 1.1 |
| FMA-UE: Fugl-Meyer Assessment-Upper Extremity section; MAL: Motor Activity Log; AOU: Amount of Use; QOM: Quality of Movement; SIS: Stroke Impact Scale; Std. Err.: Standard Error | | | | | | | | | | | | | | |
